# Physicochemical Properties and Antioxidant Capacity of Honey from Honey Bee (**Apis mellifera**): Spectrophotometric and Electrochemical Assay

**DOI:** 10.1021/acsomega.4c11602

**Published:** 2025-03-08

**Authors:** Izabela de F. Schaffel, Gabriel F. S. dos Santos, Bruna M. Damm, Ronaldo Augusto de S. Santos, José Gustavo L. Almeida, Helder C. Resende, Vanessa R. Matos, Maria Cristina Gaglianone, Emanuele Catarina da S. Oliveira, Edna Maria M. Aroucha, Rafael de Q. Ferreira

**Affiliations:** † Department of Chemistry, Federal University of Espírito Santo, 29075-910 Vitória, ES, Brazil; ‡ Department of Engineering and Environmental Sciences, Federal Rural University of the Semi-Árido, 59625-900 Mossoró, RN, Brazil; § Bee Conservation Genetics Laboratory−LaBee PPG Management and Conservation of Natural and Agrarian Ecosystems, Federal University of Viçosa, 35690-000 Florestal, MG, Brazil; ∥ Center for Biosciences and Biotechnology, North Fluminense State University Darcy Ribeiro, 28013-602 Campos dos Goytacazes, RJ, Brazil; ⊥ Federal Institute of Espírito Santo, Av. Ministro Salgado Filho, Soteco, 29106-010 Vila Velha, ES, Brazil

## Abstract

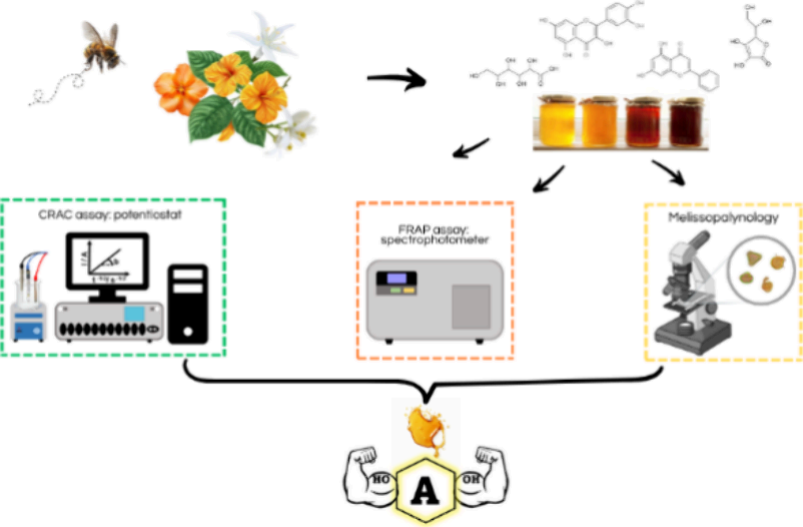

Quality parameters, physical–chemical characteristics, and antioxidant capacity are important to ensure the successful commercialization of honey. On the other hand, the presence of antioxidants in foods gives them greater added value. Thus, based on the study of total antioxidant capacity (TAC), along with the analysis of total phenolic compounds (TPC) and total flavonoid compounds (TFC), it is possible to classify honey according to its composition. First, all the samples were evaluated according to their polinic origin (melissopalynological analysis). The TAC was determined using both spectrophotometric methods (ferric reducing antioxidant power, FRAP) and electrochemical methods (ceric reducing antioxidant capacity, CRAC). All types of honey exhibited significant values for TAC, TPC, and TFC. To assess honey quality, methodologies such as moisture content, diastase activity, hydroxymethylfurfural (HMF), total acidity (TA), and water activity (*A*_w_) were employed. Among the analyzed samples, 5 and 2 honey showed high levels of moisture (20.10–20.87%), TA (65.12–65.49 mequiv kg^–1^), and HMF (>60 mg kg^–1^), surpassing the limits set by Brazilian legislation. This indicates a potentially longer shelf life or the possibility of adulteration. Furthermore, it was concluded that the color of honey may interfere with FRAP (*r* = 0.837). In this regard, the CRAC assay presents a new alternative for evaluating TAC in bee products.

## 1 Introduction

Bee honey is a product consumed worldwide, and for its commercialization, there is strict international^1^ and national^2^ legislation to verify its physical-chemical and microbiological suitability. The physical-chemical properties differ depending on the bee species, botanical origin, geographic and environmental factors at the time of extraction.^3−6^

Regarding the honey from honey bee (*Apis mellifera*), Brazilian legislation acts by the Codex standards but with few modifications, with physical-chemical parameters to assess honey maturation (reducing sugars, apparent sucrose, and moisture), deterioration (acidity, diastase activity, and furfural), and purity (solids insoluble in water, minerals).^2^ These quality concerns follow the demand in Brazil since honey is a product commonly purchased at street markets, supermarkets, or by family farmers.^7^

Floral honey can be classified as monofloral or polyfloral, with the presence of pollen from one species or various floral species, respectively.^2^ This is possible because bees cover an area within a radius of approximately 3 km in search of floral resources.^8^ Generally, monofloral honey has a higher market value compared to polyfloral honey, which is classified as wild honey.^9^

Regardless of the type of honey, the majority content of honey is the reducing sugars, glucose, and fructose, followed by water and the minority contents are organic acids, amino acids, minerals, vitamins, wax, pollen grains, phenolic compounds, pigments, and aromatic substances that contribute to its color, odor, and flavor.^10^ On the other hand, polyfloral honey may stand out as having a higher content of phenolic substances, which may reflect a greater antioxidant capacity.^11^

In addition to phenolic substances (caffeic acid, salicylic acid, quercetin, chrysin, and others), honey contains hydrogen peroxide, gluconic acid, and ascorbic acid, which can increase the antioxidant and antimicrobial properties of honey.^10,12,13^

Antioxidant compounds provide good preservation of honey, increasing oxidation stability and delaying the loss of sensory characteristics (flavor and color) and their quantity may be related to the botanical origin.^14−16^ Moreover, antioxidant compounds act similarly to a sacrificial metal in human metabolism, transferring electrons to neutralize free radicals and preventing their harmful oxidative effects, which can cause cardiovascular disease and cancer. Therefore, phenolic compounds (phenolic acids and flavonoids) have antioxidant properties, giving honey great beneficial activity, such as antimicrobial, antiviral, antiparasitic, anti-inflammatory, antineoplastic, antiulcer, antitumor, immunosuppressive action and control of cardiovascular diseases.^10,17^

In addition, studies have been combined to quantify total phenolic compounds (TPC) and total flavonoid compounds (TFC) in honey samples.^18,19^ There are several methodologies to analyze antioxidant compounds in food, such as assays to determine the total antioxidant capacity (TAC). Therefore, to assess the antioxidant capacity of honey, some studies have been reported, such as FRAP (*ferric reducing antioxidant power*), DPPH (2,2-diphenyl-1-picrylhydrazyl), and ABTS (2,2-azinobis (3-ethylbenzothiazoline-6-sulfonic acid)).^19^ These methodologies employ spectrophotometric techniques to determine TAC. Nevertheless, the colors of the analyzed samples can directly influence the absorbance values found, providing questionable results.^15,20−22^

On the other hand, the CRAC (ceric reducing antioxidant capacity) assay is based on an electrochemical technique (chronoamperometry) to determine TAC.^23^ In this methodology, the color of the sample does not act as an interferer; therefore, it may present a more reliable result. Although there are still no reports in the literature regarding its use in honey, it has great potential for analyzing the antioxidant capacity of several food matrices, such as fruit juices, sugar cane juice, and passion fruit extracts.^20^

Therefore, this study aimed to evaluate honey sold in markets and supermarkets regarding TAC and physicochemical characteristics: moisture, total acidity (TA), HMF, diastase activity, and water activity (*A*_w_). In addition to these parameters, antioxidants were assessed using the TPC and TFC techniques. Two methods were applied to evaluate the TAC: a method already known for honey samples based on spectrophotometry (FRAP assay) and reported a lack of consistency on colored or turbid samples and another not yet applied to the matrix under study based on an electrochemical technique (CRAC assay) and therefore not influenced by color, only by the electrical properties of the solution.

## 2 Material and Methods

### 2.1 Instrumentation

All aqueous solutions used in the experiments were prepared using ultrapure water (resistivity of 18 MΩ cm, 25 °C) obtained via a reverse osmosis purification system (Sartorius arium mini-Germany).

Electrochemical experiments were carried out in a borosilicate glass cell (50 mL) with an acrylic lid that accommodates the conventional three-electrode system and a small hole to facilitate the insertion of reaction solutions. The electrodes employed were a platinum bar as an auxiliary electrode (Metrohm–Netherlands), a saturated calomel KCl 3.0 mol L^–1^ (Analyzer–Brazil) as a reference electrode, and a boron-doped diamond (BDD) as a working electrode. To construct the BDD electrode, a boron-doped diamond film doped with 8000 ppm boron (NeoCoat–Switzerland) with a geometric area of 0.29 cm^2^, was fixed with tin solder on a copper-coated integrated circuit board, later coated with an epoxy resin (Araldite–Brazil) to insulate the copper and keep only the BDD film exposed. These tests were performed with an AUTOLAB PGSTAT 128N potentiostat/galvanostat (Metrohm–Netherlands) applying the NOVA 2.1.4 program interface (Metrohm–Netherlands).

The FRAP assay utilized a spectrophotometer UV–Vis DR 5000TM (HACH–USA). For the analysis of color, TPC, TFC, HMF, and diastase activity, the spectrophotometer UV-340G (GEHAKA–Brazil) was used. The moisture was determined by a manual refractometer RT-90ATC (SAMMAR–China). The *A*_w_ was determined using an ITK Wuxi Hake Apparatus electronic device (model HD-3A–China).

### 2.2 Honey Samples

Twelve samples of honey were acquired in fairs and supermarkets of Espírito Santo, in different periods of 2021 as described in Table 1. The samples were maintained in plastic packaging at room temperature (25 ± 2 °C).

**Table 1:** Classification of Honey (**Apis mellifera** L.), with Their Respective Pollens Identified by Melissopalynological Analysis and Places of Acquisition in Fairs and Supermarkets, Proceeds of the Different Regions of Espírito Santo, Brazil^a^

	identified pollens		
sample	DP^b^	AP^b^	acquisition location
1	*Eucalyptus* (71.6%)		Fundão/ES
2		*Cordia* (27.1%)	Vargem Alta/ES
3	*Coffea arabica* (50.1%)	Asteraceae (20.1%)	Domingos Martins/ES
4	*Citrus* (51.4%)		Domingos Martins/ES
5	*Myrcia* (54.7%)		Domingos Martins/ES
6		*Acacia* (40,9%)	Domingos Martins/ES
7		*Schinus* (41.2%) *Senna* (41.2%)	Domingos Martins/ES
8	*Coffea arabica* (50.64%)	*Cordia* (20.9%)	South capixaba
		*Eucalyptus* (20.9%),	
9		*Coffea arabica* (28.7%)	São Mateus/ES
10		Fabaceae *sp1* (28.2%)	São Mateus/ES
		*Coffea arabica* (16.5%)	
11		*Mimosa pudica* (42.8%)	Colatina/ES
		*Mabea* (20.5%)	
12		*Eucalyptus* (37.6%)	Ibiraçu/ES

a Identification of the apiary registry: Municipal inspection service, Espírito Santo Institute of Agricultural and Forestry Defense.

b DP-dominant pollen (>45% of the total grains in the sample); AP-accessory pollen (16–45%). Note: iiP-important isolated pollen (3–15%) and oiP-occasional isolated pollen (<3%) are presented in Table S1 (Supporting Information).

### 2.3 Melissopalynological Analysis of Honey Samples

To confirm the presence of pollen and certify if the sample is floral honey, as well as to identify the nectar sources that gave rise to the sampled honey, analyses were conducted using the acetolysis method.^24^ The microscopy slides were prepared in glycerinated gelatin and sealed with paraffin.^24^ All samples were observed under an optical microscope (model Zeizz Primo Star). Pollen identification was carried out using comparative data in the literature, defining the pollen types present in each honey sample.

The frequency of each pollen type per sample was determined, and the types were classified according to their occurrence following Louveaux et al.: dominant pollen—DP (>45% of the total grains in the sample), accessory pollen—AP (16–45%), important isolated pollen—iiP (3–15%), and occasional isolated pollen—oiP (<3%).^25^

### 2.4 Physicochemical Properties

Physicochemical analyses were performed according to the Association of Official Analytical Chemists,^26^ and the results were compared to the Brazilian and international standards.^1,2^ The moisture of the different honey samples was determined by a manual refractometer, which expresses the results in % (w/w). For TA, 10 g of honey was diluted in 75 mL of ultrapure water, followed by titration with NaOH 0.050 N, which was interrupted when the solution reached pH 8.5. The *A*_w_ was first determined by calibrating the equipment with a NaCl solution. Then, 7.5 mL of honey was added to a Petri dish to determine the dew point of the sample.^26^

HMF was determined with a clarifying agent, Carrez solutions (I and II), NaHSO_3_ 0.20%, and reading the absorbance of the solutions at 284 and 336 nm in a spectrophotometer. For the analysis of diastatic activity, a buffered solution of soluble starch and honey was incubated at 40 °C in a thermostatic bath. Subsequently, 0.5 mL from this mixture was transferred every 5 min, with 10 mL of iodine 7.0 × 10^–4^ N solution, and sample absorption at 660 nm was monitored until the absorbance value was less than 0.235. The result was expressed using the Gothe scale.^26^

### 2.5 Analysis of Antioxidant Properties

#### 2.5.1 CRAC Assay

Initially, cyclic voltammetry was used to identify the reduction potential of Ce^IV^ in the range between 0.5 and 1.75 V (100 mV s^–1^ scan rate). Moreover, to monitor the concentration of Ce^IV^ species was initially applied an open circuit potential (≈1.2 V) for 5 s, followed by a reduction potential (0.60 V) for 10 s, recording the current variation over time (Ferreira and Avaca 2008). Furthermore, to improve the analytical response of the BDD electrode, an anodic treatment (+3.0 V) for 30 s was performed, followed by a cathodic treatment (−3.0 V) for 90 s in a solution of H_2_SO_4_ 0.50 mol L^–1^, for cleaning and activating the electrode surface.^27^ This pretreatment ensured that all analyses were reliable and reproducible.

The Ce^IV^ oxidant solution was prepared at a concentration of 1.0 × 10^–3^ mol L^–1^ in H_2_SO_4_ 0.50 mol L^–1^. From this solution, a calibration curve was constructed using five different concentrations (0.20; 0.40; 0.60; 0.80, and 1.0 × 10^–3^ mol L^–1^) of the oxidant as a function of the Cottrell slopes. To determine the antioxidant capacity of the test molecule and of the honey of different flowering, 15 mL of 1.0 × 10^–3^ mol L^–1^ Ce^IV^ solution in H_2_SO_4_ 0.50 mol L^–1^, reacted for 4 min with an aliquot of 0.1 mL of the trolox stock solution and a honey stock solution (0.40 g mL^–1^ in H_2_SO_4_ 0.50 mol L^–1^). The result of the antioxidant capacity was expressed in trolox equivalent (TE).

#### 2.5.2 FRAP Assay

FRAP reagent was prepared before analysis according to the methodology described by Benzie and Strain.^28^ The FeSO_4_.7H_2_O solution was prepared at a concentration of 1.0 × 10^–3^ mol L^–1^. From this solution, dilutions were performed to five different concentrations (0.20–1.0 × 10^–3^ mol L^–1^) to construct the calibration curve. For this, was reacted 0.060 L of each dilution, along with 1.80 mL of FRAP reagent and 0.18 mL of ultrapure water. For reaction with the sample, 0.060 mL of honey solutions (0.20 g mL^–1^ in H_2_SO_4_ 0.50 mol L^–1^) of different flowering, 1.80 μL of the FRAP reagent, and 0.18 mL of ultrapure water, for 4 min at 37 °C. The intensity of the color change was measured from the absorbance at 595 nm against a blank containing only H_2_SO_4_ solution 0.50 mol L^–1^, FRAP reagent, and ultrapure water. The same procedure was performed with the stock trolox solution obtaining a final concentration of 1.0 × 10^–5^ mol L^–1^. The results of the antioxidant capacity in the honey samples were expressed in TE and mol Fe^II^ kg^–1^ of honey using the calibration curve.

#### 2.5.3 TPC Analysis

TPC analyses were performed using the Folin-Ciocalteau reagent, described by Singleton et al.^29^ Initially, 0.50 mL of honey solution (0.10 g mL^–1^ in ultrapure water) and 2.5 mL of 0.2 N Folin-Ciocalteau reagent were added. After 5 min, 2.0 mL of 75 g L^–1^ sodium carbonate solution was added. This mixture was incubated in the dark at room temperature (≈25 °C) for 2 h, and then the absorbance was measured at a wavelength of 760 nm against a blank (methanol).^30^ Subsequently, to calculate the TPC value, the data were correlated to the linear equation of the calibration curve for gallic acid (20–160 mg L^–1^), and the results were expressed in gallic acid equivalent (GAE), in other words, mg GAE 100 g^–1^ of honey.

#### 2.5.4 TFC Analysis

TFC analyses were determined according to the methodology described by Meda et al. and Ahn et al., with some adaptations.^30,31^ A solution of AlCl_3_ 2.0% diluted in methanol was prepared. 5.0 mL of AlCl_3_ (2%) was added to the same volume of honey solution (0.020 mg mL^–1^). After 10 min, the absorbance was read at a wavelength of 415 nm, using methanol as a blank. A quercetin calibration curve (0–10 mg L^–1^) was used as a standard to obtain the results. Finally, TFC was expressed in quercetin equivalent (QE), as follows: mg QE 100 g^–1^ of honey.

### 2.6 Color Analysis

The color classification was performed on the honey samples, using a spectrophotometric technique to obtain absorbance at 560 nm, using glycerin as white. The absorbance results were later converted according to the Pfund scale.^32^

### 2.7 Statistical Analysis

Tukey’s test and Pearson correlation (*r*) with their respective *p*-values, were used to evaluate the differences and correlations, respectively, between all the physicochemical parameters TAC, TPC, and TFC of honey samples, with Google Sheets Software (Google–USA) and 95% confidence level.^33^

## 3 Results and Discussion

### 3.1 Melissopalynological Analysis of Honey Samples

The melissopalynological analysis indicated a variety of pollen types present in the honey samples analyzed. The dominant pollens, defined as those with a frequency of 45% or higher, can be considered monofloral honey and labeled with a specific botanical origin.^21^ The results obtained regarding the frequency of dominant and accessory pollens (genus and family) in all the samples are presented in Tables 1 and S1 (Supporting Information).

Upon reviewing Table 1, it is evident that only samples 1, 3, 4, 5, and 8 can be described as monofloral. Sample 1 can be categorized as Eucalyptus honey (71.6%). Eucalyptus is a tree native to Australia. However, it has been widely planted in Espírito Santo and other regions of Brazil as an important activity in the rural economy, being one of the main sources of raw material for the paper and pulp industry. In Espírito Santo, in 2021, the cultivated area of Eucalyptus was 275,486 ha.^34^

Samples 3 and 8 contained dominant pollens of *Coffea arabica* at 50.1 and 50.64%, respectively. Additionally, sample 3 exhibited accessory pollen from *Asteraceae* (20.1%), while sample 8 contained *Cordia* (20.9%) and *Eucalyptus* (20.9%). The Asteraceae family is one of the most biodiverse botanical families in the world, with a wide distribution in Espírito Santo, featuring 112 genera and 268^35^ species recorded in this state. Noteworthy examples include *Baccharis dracunculifolia*, *Eremanthus erythropappus*, and *Vernonia polyanthes*, which are of apicultural interest in the region. The genus *Cordia* is also represented by species of apicultural interest, especially *Cordia trichotoma*.^36,37^

Sample 4 was characterized by the predominant presence of *Citrus* pollen (51.4%), a botanical genus commonly found in crops in the region.^34^ Sample 5 exhibited a dominant pollen type of *Myrcia* (54.7%), a botanical genus belonging to the Myrtaceae family, widely distributed in Brazil and with records of 103 different species in Espírito Santo.^35^

The remaining samples did not exhibit dominant pollen types and were classified as multifloral honey samples. The accessory pollens identified in samples 2, 6, 7, 10, and 11 represent species found in the Atlantic Forest of Espírito Santo, such as *Mimosa pudica*, species of the botanical genera *Cordia* spp., *Acacia* spp., *Schinus* spp., *Senna* spp., *Mabea* spp., and the botanical family Fabaceae.^35^ The accessory pollens identified in samples 9 and 12 represent cultivated plants in Espírito Santo, namely *Coffea arabica* and *Eucalyptus* spp. Both the pollen types of native and cultivated plants found in all samples represent plant species of apicultural interest for honey production.

### 3.2 Physicochemical Properties

All physicochemical parameters (moisture, TA, HMF, diastase, and *A*_w_) obtained for the honey analyzed are presented in Table 2. The moisture content ranged from 16.87 to 21.03%. Sample 2 (20.10 ± 0.10%), sample 5 (20.87 ± 0.15%), sample 11 (21.03 ± 0.06%), and sample 12 (20.57 ± 0.06%) exceeded the maximum limit (20%) established by Brazilian and international standards. Several factors can contribute to the moisture increase, such as honey harvesting coinciding with a rainy season, harvesting of unripe honey, unsuitable honey storage and storage places, and the moisture of the air surrounding the beehives.^38^ Sample 7 had a significantly lower content (16.87 ± 0.12%) than all other kinds of honey.

**Table 2:** Results of the Analysis Physicochemical Analysis (Moisture, TA, HMF, Diastase Activity, and *A*_w_) Found for the Respective Samples of **Apis mellifera** Honey from Different Flowering Areas in Espírito Santo, Brazil^a^

sample	moisture (%)	*A*_W_	TA (meq kg^–1^)	diastase activity (°Gothe)	HMF (mg kg^–1^)
1	19.53 ± 0.06^d^	0.62 ± 0.02^a^	49.51 ± 0.14^b^	13.22 ± 0.80^ef^	76.17 ± 1.14^c^
2	20.10 ± 0.10^c^	0.63 ± 0.03^a^	65.12 ± 0.10^a^	11.23 ± 0.77^fg^	103.04 ± 3.65^a^
3	17.87 ± 0.12^f^	0.54 ± 0.05^a^	39.23 ± 0.09^d^	50.24 ± 1.6^a^	16.39 ± 0.44^h^
4	18.13 ± 0.15^f^	0.54 ± 0.03^a^	27.24 ± 0.06^h^	9.41 ± 0.51^g^	43.64 ± 1.11^e^
5	20.87 ± 0.15^ab^	0.61 ± 0.02^a^	65.49 ± 0.45^a^	13.78 ± 0.46^e^	85.32 ± 0.92^b^
6	18.00 ± 0.10^f^	0.57 ± 0.05^a^	29.90 ± 0.68^g^	14.96 ± 0.45^e^	47.99 ± 0.47^d^
7	16.87 ± 0.12^g^	0.55 ± 0.07^a^	25.82 ± 0.65^h^	48.83 ± 0.70^a^	10.47 ± 0.68^i^
8	18.57 ± 0.06^e^	0.59 ± 0.03^a^	34.92 ± 0.89^f^	29.82 ± 0.54^b^	25.65 ± 1.13^fg^
9	19.87 ± 0.12^c^	0.60 ± 0.03^a^	45.32 ± 0.90^c^	28.33 ± 0.46^bc^	28.59 ± 0.74^f^
10	18.50 ± 0.10^e^	0.60 ± 0.04^a^	37.70 ± 0.91^de^	27.56 ± 0.46^c^	24.59 ± 0.50^g^
11	21.03 ± 0.06^a^	0.64 ± 0.03^a^	36.29 ± 1.03^ef^	17.10 ± 0.20^d^	18.22 ± 0.91^h^
12	20.57 ± 0.06^b^	0.64 ± 0.05^a^	35.80 ± 0.62^f^	26.62 ± 0.46^c^	25.27 ± 1.13^fg^
Brazil 2000	<20		<50	>8 or 3	<60
Codex 2019	<20		<50	>8 or 3	<80^b^

a Results are expressed as the mean ± standard deviation (SD) of the triplicates performed. Means followed by the same letter in the column do not differ according to Tukey’s test at *p* ≤ 0.05.

b Countries or regions with tropical ambient temperatures.

For TA, most kinds of honey showed values within the parameters needed by both legislations (<50 mequiv kg^–1^), but in samples 2 and 5, honey showed values outside the standards.^1,2^ These samples are susceptible to the fermentation process because they presented moisture and TA above the needed standards. The fermentation of honey occurs during storage due to the activity of osmotolerant yeast. This process can synthesize ethanol and carbon dioxide. Moreover, alcohol may be oxidized into acetic acid and water, thereby contributing to the sour taste of honey.^39^

Diastase activity varied between 9.41 and 50.24°Gothe, therefore all samples showed values that meet the standards established by Brazilian legislation and international normative, with values above 8°Gothe.^1,2^ Diastase is an enzyme (α-amylase) whose function is to hydrolyze starch. This enzyme is formed in bees mainly by the hypopharyngeal glands and can be found in pollen grains in low proportions. Diastatic activity is a parameter that indicates the quality of honey, as its altered levels are related to storage conditions and inadequate heating, and when its value is equal to zero, it may indicate adulteration.^40^

In this study, the HMF content in the honey samples ranged from 10.47 to 103.04 mg kg^–1^. However, three analyzed samples (1, 2, and 5) showed values outside those needed by Brazilian legislation (<60 mg kg^–1^). For international standards, samples 2 and 5 exceeded the allowed value (<80 mg kg^–1^). This parameter is the only one that differs between the Brazilian legislation and the Codex Alimentarius.^1,2^ The commission of Codex considers that tropical regions (e.g., Latin America, Africa), which have an average climate with temperatures higher than others, produce more HMF. The samples that showed values outside the standards suggest inadequate handling, such as the honey overheating or excessive storage. Thus, HMF is an indicator of honey freshness, and high levels indicate the degradation of sugars in honey, formed by two reactions: acid-catalyzed degradation of hexose; and decomposition of 3-deoxy-osone in the Maillard reaction.^41^

*A*_w_ ranged from 0.54 to 0.64, therefore, it can be considered that the analyzed samples do not favor the growth of yeasts and fungi. *A*_w_ is related to the amount of water available for the growth of microorganisms, such as fungi, yeasts, and bacteria. For honey, this parameter is not a requirement of current legislation, however, it is an important indicator of shelf life and possible degradation and fermentation reactions and should be in the normative documents. Yeast and mold growth are disadvantaged at *A*_w_ below 0.85 and 0.70, respectively. Consequently, it is possible to consider a stable or still dehydrated food when the water activity is less than 0.60.^42^

### 3.3 Color Analysis

Honey can be classified by the Pfund scale, and values between 50 and 85 represent light amber honey. With the increase in the Pfund value (85–114), the honey becomes darker and is classified as amber. For values above 114, honey is classified as dark amber. The color of honey may be related to its botanical origin.^10^ In our analyses (Table 3), the darkest honey was sample 5 (Pfund = 620.15), while sample 10 resulted in the lightest product (Pfund = 64.92). Sample 4 of *Citrus* honey has the second clearest value on the Pfund scale. These values are in accordance with the literature, which shows the main characteristics of citrus flower honey, besides the light color, have an intense odor, delicate flavor, and fine crystallization.^43^

**Table 3:** Results of the Analysis of Antioxidant Capacity (CRAC and FRAP), TPC, TFC, and Color Found for the Respective Samples of **Apis mellifera** Honey from Different Flowering Areas in Espírito Santo, Brazil^a^

		FRAP				coloring	
sample	CRAC (TE)	(TE)	(10^–3^ mol Fe^II^ kg^–1^)	TPC (mg GAE 100 g^–1^)	TFC (mg QE 100 g^–1^)	Pfund	color
1	0.89 ± 0.05^d^	0.606 ± 0.025^d^	1.778 ± 0.073^d^	62.90 ± 1.06^i^	1.65 ± 0.09^f^	210.50	dark amber
2	1.08 ± 0.07^d^	0.722 ± 0.035^c^	2.118 ± 0.104^c^	115.68 ± 1.07^e^	2.32 ± 0.32^de^	275.12	dark amber
3	1.20 ± 0.08^cd^	0.397 ± 0.009^e^	1.166 ± 0.026^e^	161.03 ± 2.84^b^	0.92 ± 0.03^g^	93.89	light amber
4	1.71 ± 0.15^bc^	0.388 ± 0.003^e^	1.140 ± 0.008^e^	53.97 ± 0.22^j^	0.64 ± 0.07^g^	82.74	light amber
5	3.09 ± 0.21^a^	0.993 ± 0.028^a^	2.913 ± 0.083^a^	88.44 ± 0.30^g^	2.93 ± 0.05^bc^	620.15	dark amber
6	1.94 ± 0.25^b^	0.274 ± 0.006^f^	0.804 ± 0.018^f^	47.23 ± 1.46^k^	0.69 ± 0.05^g^	75.32	light amber
7	1.20 ± 0.05^cd^	0.706 ± 0.048^c^	2.073 ± 0.142^c^	129.58 ± 0.48^d^	6.64 ± 0.44^a^	422.67	dark amber
8	1.50 ± 0.09^c^	0.379 ± 0.004^e^	1.111 ± 0.011^e^	145.88 ± 1.05^c^	1.08 ± 0.09^g^	118.40	amber
9	1.25 ± 0.20^cd^	0.562 ± 0.018^d^	1.649 ± 0.054^d^	92.66 ± 0.89^f^	2.51 ± 0.03^cd^	126.57	amber
10	1.34 ± 0.06^cdi^	0.401 ± 0.018^e^	1.178 ± 0.052^e^	92.01 ± 0.85^fg^	1.79 ± 0.09^ef^	64.92	extra light amber
11	1.17 ± 0.05^cd^	0.744 ± 0.004^bc^	2.185 ± 0.0011^bc^	68.17 ± 1.42^h^	2.49 ± 0.06^cd^	609.38	dark amber
12	1.21 ± 0.09^cd^	0.798 ± 0.016^b^	2.342 ± 0.046^b^	245.60 ± 1.58^a^	3.26 ± 0.27^b^	210.87	dark amber

a Results are expressed as the mean ± standard deviation (SD) of the triplicates performed. Means followed by the same letter in the column do not differ according to Tukey’s test at *p* ≤ 0.05.

### 3.4 TPC Analysis

Phenolic content is compounds that can influence the color of honey and its functional properties.^44^ The determination of total phenolic compounds by the Folin-Ciocalteau method for the honey samples is specified in Table 3. The results showed significant differences among investigated samples due to the different flowerings analyzed, ranging from 47.23 to 245.60 mg GAE 100 g^–1^ of honey, referring to sample 6 and sample 5, respectively. These two samples, with the minimum and maximum values, are presented in the color test within the Pfund scale light amber and dark amber, respectively. However, sample 3 with a light amber color had an expressively high TPC value of 161.03 mg GAE 100 g^–1^ of honey, demonstrating that the phenolics present in coffee honey may not have a characteristic color.

Coffee honey samples had the highest values (sample 3, 161.03 mg GAE 100 g^–1^ of honey and sample 8, 154.88 mg GAE 100 g^–1^ of honey) after honey with a predominance of Eucalyptus flowering as accessory pollen (sample 12, 245.60 mg GAE 100 g^–1^ of honey). The characterization of phenolic compounds in coffee honey is known in the literature, and the compounds present in higher concentrations most of the time are gallic acid, ferulic acid, and chlorogenic acid.^17^ The TPC values of this study are considerably higher than those reported for other studies from south Brazil.^18^

The results showed a weak and negative Pearson correlation (*r* = −0.0080) between the phenolic content and the color in the samples of capixaba honey from the flowering under study (Table 4).

**Table 4:**
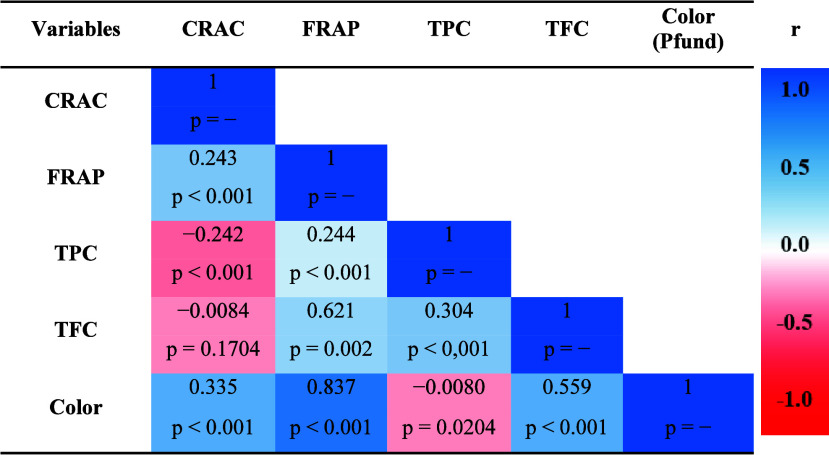
Pearson Correlation Coefficients (*r*) and Probability Values (*p*) of Antioxidant Capacity (CRAC and FRAP), TPC, TFC, and Pfund Staining Found in Honey Samples of **Apis mellifera** from Different Flowering in Espírito Santo, Brazil

### 3.5 TFC Analysis

The TPC analysis determines the amount of total phenolic compounds. Meanwhile, the TFC analysis is a more selective methodology to quantify only the flavonoids present since flavonoids are a class of phenolic compounds. Thus, TPC values are greater than TFC.

The total flavonoid compounds of the analyzed honey ranged from 0.64 to 6.64 mg of QE 100 g^–1^ of honey (Table 3). Sample 7 (AP: *Schinus* 41.2% and *Senna* 41.2%), considered dark, presented the third-highest TPC value (129.58 mg GAE 100 g^–1^ of honey) and the highest TFC (6.64 mg of QE 100 g^–1^ of honey). These results agree with the work published by Pena Júnior et al., who carried out a similar study in flowering plants in Minas Gerais–Brazil, for which high values of TPC (101.67 mg of GAE 100 g^–1^ of honey) and TFC (18.94 mg of QE 100 g^–1^ of honey) were observed of aroeira honey (*Schinus*) when compared to other honey flowering.^5^

Among the samples examined, *Myrcia* (54.7%) honey had the second highest TFC values, 2.93 mg of QE 100 g^–1^ for sample 5. There are several species within the gender *Myrcia*, but most studies have focused on phenolic compounds and flavonoids. Notably, a review conducted by Stefanello, Pascoal, and Salvador highlighted essential oils from various parts of the plant (flowers, leaves, and stems) and their complex bioactive compounds, including monoterpenes and sesquiterpenes, which have been attributed with cytotoxic, antioxidant, larvicidal, antinociceptive, anti-inflammatory, and analgesic properties.^45^ Consequently, some of these compounds may be transferred to honey through pollen, imparting similar properties. In a study by Silva et al., among seven honey samples, the sample with 77.6% *Myrcia* pollen stood out with the second-highest total flavonoid content (64.0 ± 0.03 mg GAE g^–1^), demonstrating that honey with a predominance of *Myrcia* has a notable amount of flavonoids, which imparts antioxidant properties to the honey and, consequently, health benefits.^46^

Sample 4 identified as *Citrus* flower had the lowest TFC value (0.64 mg of QE 100 g^–1^ of honey) and one of the lowest TPC values (53.97 mg of GAE 100 g^–1^ of honey). However, even with low levels of TPC and TFC, several compounds have already been identified as naringenin, caffeic acid, luteolin, hesperetin, chrysin, galangin, quercetin, kaempferol, among others in the orange honey (*Citrus*).^47^

Among the analyzed samples, two have predominance the coffee flowering of different regions of Espírito Santo and with harvests carried out in various periods of the year 2021 (samples 3 and 8). Although the samples have a predominance of the same flowering, we can observe the difference in the values of TPC (161.03 and 145.88 mg of GAE 100 g^–1^ of honey, respectively) and TFC (0.92 and 1.08 mg of QE 100 g^–1^ of honey, respectively). This is due to the differences in accessory pollens, in which sample 3 has as accessory pollen *Asteraceae* (20.1%) and sample 8 *Cordia* (20.9%) with *Eucalyptus* (20.9%), which can lead to different chemical properties.^8^ The correlation coefficient obtained was moderately positive for TFC to TPC (*r* = 0.304) and color (*r* = 0.559), as shown in Table 4.

### 3.6 Determination of TAC

#### 3.6.1 FRAP Assay

With the results obtained from the FRAP assay, it is possible to classify the honey samples in the following order of antioxidant capacity: 6 < 8 < 4 < 3 < 10 < 9 < 1 < 7 < 2 < 11 < 12 < 5. FRAP values ranged from 0.274 to 0.993 TE for sample 6 DP *Myrcia* (54.7%) and sample 5 AP *Acacia* (40,9%) flowering, respectively.

Honey samples present a complex composition, reaching more than 180 compounds already identified.^10^ According to some studies, there is often a positive correlation between those parameters (TPC, TFC, and TAC). However, when analyzing the data in detail, it is possible to notice that there are exceptions for honey that do not follow this relationship. An example is a study reported by Pena Júnior et al., who evaluated the TAC by DPPH of two samples of aroeira flowering, one resulted in a higher antioxidant capacity (EC_50_ = 11.30 mg mL^–1^) than the other (EC_50_ = 15.00 mg mL^–1^).^5^ However, the sample with a lower antioxidant capacity showed higher values of TPC and TFC. Likewise in the same study, the caiaté flowering resulted in a light amber color and obtained a higher TAC (EC_50_ = 18.27 mg mL^–1^) when compared to the amber-colored betony flowering (EC_50_ = 31.49 mg mL^–1^). It should be noted that for the TAC assay used in this study (DPPH), the results are expressed in EC_50_ (amount of antioxidant needed to reduce the initial concentration of the DPPH radical by 50%), and the lower this value, the greater the capacity sample antioxidant.^5^ Although it is possible to correlate the color with its chemical composition (TPC, TFC, and TAC), this is not a statement. The pigments of honey could be related to carotenoid, honey’s age, botanical origin, contact with metallic materials during storage, and postharvest handling.^48^

The FRAP assay showed a low correlation between TPC and TFC. However, a high correlation with color (*r* = 0.837) was obtained. This high correlation may be related to the considerable interference of coloration in the FRAP assay already discussed in some previous studies, which is one of the disadvantages of using spectrophotometric assays in colored or turbid samples.^17^ Some authors also show that this relationship is not exact. For example, in the study reported by Arenhart and Fogaça, it was identified that darker wines had low TAC.^48^ Thus, these antioxidant compounds not always are associated with color.

#### 3.6.2 CRAC assay

**Figure 1:**
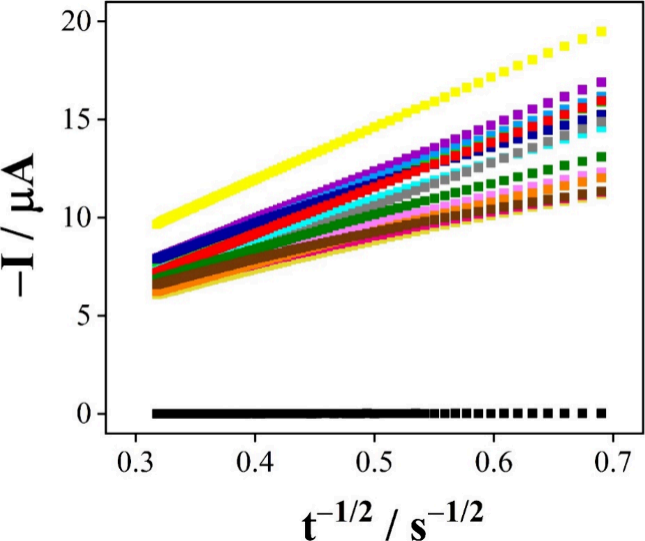
Relation of *I* with *t*^–1/2^ from the Cottrell equation after reaction with kinds of honey from different flowering ((violet square) 1: Wild; (light blue square) 2: Wild; (light green square) 3: Coffee; (dark blue square) 4: Orange; (brown square) 5: Camara; (cyan square) 6: Capixingui; (gray square) 7: Aroeira; (magenta square) 8: Coffee; (pale yellow square) 9: Coffee; (pink square) 10: Coffee with macadamia; (orange square) 11: Mamoninha; (dark green square) 12: Camara, (red square) trolox, (black square) blank: H_2_SO_4_ 0.5 mol L^–1^ and (yellow square) Ce^IV^ 1.0 × 10^–3^ mol L^–1^.

The CRAC value for each honey flowering, and the TE, were calculated using the slope values of the Cottrell curves (b), which are presented in Figure 1, and the results are listed in Table 3.

From Figure 1, it is possible to notice that most of the honey presented straight lines with a lower slope than that obtained by trolox (red line), which suggests that this honey can reduce Ce^IV^ more efficiently than the molecule of the test. Therefore, the sample with the highest antioxidant capacity was 5 identified as with a predominance of pollen of the gender *Myrcia* (TE = 3.09), represented by the brown line in Figure 1. In addition, it is worth noting that this botanical origin, resulted in the second highest value of FRAP (TE = 0.993) and second highest value of flavonoids (2.93 mg QE 100 g^–1^ of honey). However, this flowering was outside the parameters required by legislation regarding moisture, HMF, and TA analysis.

Thus, it is crucial to emphasize that trolox is the water-soluble equivalent of vitamin E. Therefore, all TE values greater than 1.0 indicate that the analyzed sample has an antioxidant capacity superior to vitamin E. The reason that may have influenced the high antioxidant capacity of sample 5 with a predominance of *Myrcia* pollen (54.7%) is the various compounds that may be present, such as those found in the plant and flowers that can be carried into the honey, including some monoterpenes and sesquiterpenes. As previously explored in essential oils by Stefanello et al. and in honey itself in the study by Silva et al., where the sample with predominant *Myrcia* stood out in terms of total flavonoid content (TFC), which in turn imparts antioxidant properties to the honey.^45,46^

With the results obtained from the CRAC assay, it is possible to classify the honey in the following order of antioxidant capacity: 1 < 2 < 11 < 7 < 3 < 12 < 9 < 10 < 8 < 4 < 6 < 5. Samples 7 and 3 showed no significant difference between the values of antioxidant capacity by the CRAC assay.

The Pearson correlation between the CRAC and FRAP assays was relatively low (*r* = 0.243). As with the FRAP assay, CRAC showed no significant correlation with TPC and TFC. However, this is because honey contains other substances that may contribute to its antiradical action, including enzymes, amino acids, proteins, vitamins, and organic acids. Therefore, the antioxidant capacity cannot be attributed exclusively to the presence of phenolic compounds.^49^

The CRAC assay is an electrochemical methodology (chronoamperometry), and it is unaffected by the presence of colored substances, in contrast to the FRAP assay, which demonstrated a modest association with color. Consequently, the CRAC assay could be more suited for determining the antioxidant capacity of colored honey samples.

## 4 Conclusions

The parameters set by Brazilian and international standards are crucial for classifying honey quality. In this study, honey samples were assessed for moisture, HMF, diastase activity, TA, and *A*_w_. Out of 12 samples, four (samples 2, 5, 11, and 12) exceeded the moisture limit set by both Brazilian and international standards. For HMF and TA, three samples (1, 2, and 5) had values outside the acceptable range according to Brazilian legislation, with samples 2 and 5 also exceeding international standards.

The study of antioxidant content in honey from various flowers revealed significant variability in these compounds. All samples showed high values of TAC, TFC, and TPC. Honey from the *Myrcia* genus (sample 5) was notable for having the highest CRAC value (TE = 3.09), the second highest FRAP value (TE = 0.993), the second highest flavonoid content (2.93 mg QE 100 g^–1^ of honey), and the darkest color on the Pfund scale. Pearson’s correlation analysis highlighted the strongest correlation between staining and the FRAP assay (*r* = 0.837), suggesting potential limitations of the FRAP assay with colored samples. Conversely, the CRAC assay showed a moderate correlation (*r* = 0.335), indicating that electrochemical techniques are less affected by sample coloration. Thus, the CRAC assay is recommended for evaluating honey’s antioxidant capacity across different flowers and colors. Combining the CRAC assay with TFC and TPC analysis provides a comprehensive evaluation of honey’s antioxidants.

The study also suggests incorporating the *A*_w_ methodology into legislation to monitor microorganism growth and antioxidant levels, as honey is often consumed for its therapeutic properties. Overall, this research offers valuable insights into the antioxidant characteristics of honey from various flowers and regions in Espírito Santo and underscores the need for rigorous inspection of honey in commercial settings.

## Supporting Information Available

Results of the melissopalynology analysis, showing all the identified pollens (dominant (DP), accessory (AP), important isolated pollen (iiP), and occasional isolated pollen (oiP)), with the respective taxon family and frequency (%) (PDF)
