# Supplementary material for: Physicochemical Properties and Antioxidant Capacity of Honey from Honey Bee (Apis mellifera): Spectrophotometric and Electrochemical Assay
Source: ACS Omega. 2025 Mar 8;10(10):10653–61. doi: 10.1021/acsomega.4c11602 (PMC11923845; doi:10.1021/acsomega.4c11602)
Supplement: Supplementary file 1 — ao4c11602_si_001.pdf [file ao4c11602_si_001.pdf]

Physicochemical properties and antioxidant capacity of honey from honey bee (Apis mellifera): spectrophotometric and electrochemical assay

Izabela de F. Schaffel[1], Gabriel F. S. dos Santos[1], Bruna M. Damm[1], Ronaldo Augusto de S. Santos[1], José Gustavo L. Almeida[2], Helder C. Resende[3], Vanessa R. Matos[4], Maria Cristina Gaglianone[4], Emanuele Catarina da S. Oliveira[1,5], Edna Maria M. Aroucha[2], Rafael de Q. Ferreira[1 *].

[1]Department of Chemistry, Federal University of Espírito Santo, 29075-910 Vitória – ES

[2]Department of Engineering and Environmental Sciences, Federal Rural University of the Semi-Árido, 59625-900 Mossoró – RN, Brazil

[3]Bee Conservation Genetics Laboratory – LaBee PPG Management and Conservation of Natural and Agrarian Ecosystems, Federal University of Viçosa, 35690-000 Florestal – MG, Brazil

[4]Center for biosciences and biotechnology, North Fluminense State University Darcy Ribeiro, 28013-602 Campos dos Goytacazes – RJ, Brazil.

[5]Federal Institute of Espírito Santo, Av. Ministro Salgado Filho, Soteco, Vila Velha, 29106-010, Espírito Santo, Brazil.

[*]Corresponding author: rafael.q.ferreira@ufes.br; Department of Chemistry, Federal University of Espírito Santo, 29075-910 Vitória – ES, Brazil; Tel.: +55 27 31455417; fax: +55 27 40092826.

Supplementary Material

Table S1 contains the relevant information regarding the results of the melissopalynology analysis, showing all the pollen identified: dominant (DP) and accessory (AP).

Table S1 Results of melissopalynology analysis in relation to the pollen types and taxon family of dominant (DP) and accessory (AP) pollens

Sample	Flowering (label)*	Pollen Types	Taxon Family	Frequency (%)	Classification*
1	Wild	*Eucalyptus*	Myrtaceae	71.63	DP
		Cordia	Boraginaceae	9.93	iiP
		Myrcia	Myrtaceae	4.96	iiP
		Acacia	Fabaceae	3.55	iiP
		Mimosa sp1	Fabaceae	3.55	iiP
		Machaerium	Fabaceae	1.42	ioP
		Mimosa pudica	Fabaceae	1.42	ioP
		Mimosa sp2	Fabaceae	1.42	ioP
		Poaceae	Poaceae	1.42	ioP
		Schinus	Anacardiaceae	0.71	ioP
2	Wild	*Cordia*	Boraginaceae	27.18	AP
		Fabaceae sp2	Fabaceae	13.59	iiP
		Poaceae	Poaceae	6.80	iiP
		Fabaceae sp1	Fabaceae	5.83	iiP
		Acacia	Fabaceae	4.85	iiP
		Asteraceae sp1	Asteraceae	3.88	iiP
		Miconia	Melastomataceae	3.88	iiP
		Eucalyptus	Myrtaceae	3.88	iiP
		Rubiaceae sp2	Rubiaceae	3.88	iiP
		Schinus	Anacardiaceae	2.91	ioP
		Diospyros	Ebernaceae	2.91	ioP
		Inga	Fabaceae	2.91	ioP
		Rubiaceae sp1	Rubiaceae	2.91	ioP
		Syagrus	Arecaceae	1.94	ioP
		Asteraceae sp2	Asteraceae	1.94	ioP
		Mimosa sp1	Fabaceae	1.94	ioP
		Cedrela	Meliaceae	1.94	ioP
		Ficus	Moraceae	1.94	ioP
		Croton	Euphorbiaceae	0.97	ioP
		Euphorbia	Euphorbiaceae	0.97	ioP
		Mimosa sp2	Fabaceae	0.97	ioP
		Poaceae sp2	Poaceae	0.97	ioP
		Cecropia	Urticaceae	0.97	ioP
3	Coffee	*Coffea arabica*	Rubiaceae	50.13	DP
		Asteraceae sp1	Asteraceae	20.16	AP
		Connarus	Connaraceae	6.72	iiP
		Senna	Fabaceae	5.94	iiP
		Fabaceae sp3	Fabaceae	3.36	iiP
		Cordia	Boraginaceae	1.55	ioP
		Calophyllaceae	Calophyllaceae	1.55	ioP
		Euphorbiaceae	Euphorbiaceae	1.55	ioP
		Eucalyptus	Myrtaceae	1,29	ioP
		Erythroxylum	Erythroxylaceae	1.03	ioP
		Cedrela	Meliaceae	1.03	ioP
		Rubiaceae sp1	Rubiaceae	1.03	ioP
		Diospyros	Ebernaceae	0.78	ioP
		Acacia	Fabaceae	0.78	ioP
		Bignoniaceae	Bignoniaceae	0.52	ioP
		Croton	Euphorbiaceae	0.52	ioP
		Fabaceae sp1	Fabaceae	0.52	ioP
		Schinus	Anacardiaceae	0.52	ioP
		Fabaceae sp2	Fabaceae	0.26	ioP

		Mimosa sp1	Fabaceae	0.26	ioP
		Eugenia	Myrtaceae	0.26	ioP
		Rubiaceae	Rubiaceae	0.26	ioP
4	Orange	*Citrus*	Rutaceae	51.41	DP
		Cecropia	Urticaceae	14.12	iiP
		Euphorbiaceae	Euphorbiaceae	10.17	iiP
		Commelinaceae	Commelinaceae	6.78	iiP
		Eucalyptus	Myrtaceae	6.21	iiP
		Arecaceae	Arecaceae	4.52	iiP
		Hyptis	Lamiaceae	3.39	iiP
		Bignoniaceae	Bignoniaceae	2.26	ioP
		Asteraceae sp1	Asteraceae	0.56	ioP
		Mimosa	Fabaceae	0.56	ioP
5	Camara	*Myrcia*	Myrtaceae	54.74	DP
		Mimosa pudica	Fabaceae	10.53	iiP
		Aegiphila	Verbenaceae	8.42	iiP
		Schinus	Anacardiaceae	7.37	iiP
		Poaceae	Poaceae	6.32	iiP
		Inga	Fabaceae	5.26	iiP
		Mimosa	Fabaceae	3.16	iiP
		Lantana	Verbenaceae	3.16	iiP
		Bauhinia	Fabaceae	1.05	ioP
6	Capixingui	*Acacia*	Fabaceae	40.96	AP
		Cedrela	Meliaceae	9.04	iiP
		Senna	Fabaceae	6.02	iiP
		Sapium	Euphorbiaceae	4.34	iiP
		Rubiaceae	Rubiaceae	3.98	iiP
		Inga	Fabaceae	3.61	iiP
		Asteraceae sp1	Asteraceae	3.37	iiP
		Arecaceae	Arecaceae	3.25	iiP
		Connarus	Connaraceae	3.13	iiP
		Eucalyptus	Myrtaceae	2.77	ioP
		Croton	Euphorbiaceae	2.65	ioP
		Cecropia	Urticaceae	2.53	ioP
		Commelinaceae	Commelinaceae	2.17	ioP
		Citrus	Rutaceae	1.81	ioP
		Vernonanthura sp1	Asteraceae	1.69	ioP
		Coffea arabica	Rubiaceae	1,69	ioP
		Cordia	Boraginaceae	1.57	ioP
		Chamaecrista	Fabaceae	1.45	ioP
		Asteraceae sp2	Asteraceae	1.20	ioP
		Fabaceae sp1	Fabaceae	0.60	ioP
		Delonix regia	Fabaceae	0.48	ioP
		Fabaceae sp2	Fabaceae	0.36	ioP
		Lantana	Verbenaceae	0.36	ioP
		Asteraceae sp3	Asteraceae	0.24	ioP
		Vernonanthura sp2	Asteraceae	0.12	ioP
		Vernonanthura sp3	Asteraceae	0.12	ioP
		Vernonanthura sp4	Asteraceae	0.12	ioP
		Vernonanthura sp5	Asteraceae	0.12	ioP
		Vernonanthura sp6	Asteraceae	0.12	ioP
		Euphorbiaceae	Euphorbiaceae	0.12	ioP
7	Aroeira	*Schinus*	Anacardiaceea	41.29	AP
		Senna	Fabaceae	41.29	AP
		Mimosa pudica	Fabaceae	3.17	iiP
		Poaceae	Poaceae	3.17	iiP
		Asteraceae sp1	Asteraceae	2.41	ioP
		Asteraceae sp2	Asteraceae	2.20	ioP
		Eucalyptus	Myrtaceae	1.65	ioP
		Sapium	Euphorbiaceae	1.51	ioP
		Vernonanthura	Asteraceae	0.55	ioP
		Mimosa	Fabaceae	0.55	ioP
		Miconia	Melastomataceae	0.48	ioP

		Cecropia	Urticaceae	0.48	ioP
		Alternanthera	Amaranthaceae	0.34	ioP
		Elephantopus	Asteraceae	0.28	ioP
		Croton	Euphorbiaceae	0.21	ioP
		Arecaceae	Arecaceae	0.14	ioP
		Ludwigia	Onagraceae	0.14	ioP
		Hyptis	Lamiaceae	0.07	ioP
		Serjania	Sapindaceae	0.07	ioP
8	Coffee	*Coffea arabica*	Rubiaceae	50.64	DP
		Cordia	Boraginaceae	20.94	AP
		Eucalyptus	Myrtaceae	20.73	AP
		Commelinaceae	Commelinaceae	1.62	ioP
		Asteraceae sp1	Asteraceae	1.28	ioP
		Cedrela	Meliaceae	1.07	ioP
		Citrus	Rutaceae	0.81	ioP
		Arecaceae	Arecaceae	0.64	ioP
		Vernonanthura sp1	Asteraceae	0.38	ioP
		Bignoniaceae	Bignoniaceae	0.34	ioP
		Cecropia	Urticaceae	0.30	ioP
		Erythroxylum	Erythroxylaceae	0.26	ioP
		Machaerium	Fabaceae	0.17	ioP
		Syagrus	Arecaceae	0.13	ioP
		Caryophyllaceae	Caryophyllaceae	0.13	ioP
		Acacia	Fabaceae	0.09	ioP
		Mimosa sp1	Fabaceae	0.09	ioP
		Borreria	Rubiaceae	0.09	ioP
		Schinus	Anacardiaceae	0.09	ioP
		Asteraceae sp2	Asteraceae	0.04	ioP
		Vernonanthura sp2	Asteraceae	0.04	ioP
		Vernonanthura sp3	Asteraceae	0.04	ioP
		Mimosa sp2	Fabaceae	0.04	ioP
		Hyptis	Lamiaceae	0.04	ioP
9	Coffee	*Coffea arabica*	Rubiaceae	28.78	AP
		Cordia	Boraginaceae	13.80	iiP
		Paullinia	Sapindaceae	13.07	iiP
		Poaceae	Poaceae	10.13	iiP
		Eucalyptus	Myrtaceae	7.78	iiP
		Schinus	Anacardiaceae	5.29	iiP
		Commelinaceae	Commelinaceae	4.85	iiP
		Syagrys	Arecaceae	3.38	iiP
		Asteraceae sp1	Asteraceae	2.20	ioP
		Protium	Burseraceae	2.06	ioP
		Mimosa	Fabaceae	1.76	ioP
		Borreria	Rubiaceae	1.76	ioP
		Machaerium	Fabaceae	1.62	ioP
		Ficus	Moraceae	1.32	ioP
		Fabaceae sp1	Fabaceae	0.88	ioP
		Senna	Fabaceae	0.59	ioP
		Mouriri	Melastomataceae	0.29	ioP
		Psychotria	Rubiaceae	0.29	ioP
		Bauhinia	Fabaceae	0.15	ioP
10	Coffee and macadamiad	*Fabaceae sp1*	Fabaceae	28.22	AP
		Coffea arabica	Rubiaceae	16.56	AP
		Connarus	Connaraceae	10.43	iiP
		Euphorbiaceae	Euphorbiaceae	7.98	iiP
		Cordia	Boraginaceae	6.13	iiP
		Fabaceae sp3	Fabaceae	6.13	iiP
		Syagrus	Arecaceae	4.29	iiP
		Macadamia	Proteaceae	4.29	iiP
		Bignoniaceae	Bignoniaceae	3.68	iiP
		Eucalyptus	Myrtaceae	3.68	iiP
		Schinus	Anacardiaceae	2.45	ioP
		Erythroxyllum	Erythroxylaceae	2.45	ioP

		Cedrela	Meliaceae	1.84	ioP
		Fabaceae sp2	Fabaceae	1.23	ioP
		Polygonum	Polygonaceae	0.61	ioP
11	Mamoninha	*Mimosa pudica*	Fabaceae	42.86	AP
		Mabea	Euphorbiaceae	20.55	AP
		Poaceae	Poaceae	8.41	iiP
		Cordia	Boraginaceae	5.09	iiP
		Ficus	Moraceae	3.72	iiP
		Myrcia	Myrtaceae	3.13	iiP
		Euphorbiaceae	Euphorbiaceae	2.94	ioP
		Cecropia	Urticaceae	2.94	ioP
		Mimosa	Fabaceae	2.74	ioP
		Asteraceae sp1	Asteraceae	2.54	ioP
		Psychotria	Rubiaceae	2.35	ioP
		Asteraceae sp2	Asteraceae	1.57	ioP
		Schinus	Anacardiaceae	0.78	ioP
		Machaerium	Fabaceae	0.39	ioP
12	Camara	*Eucalyptus*	Myrtaceae	37.66	AP
		Euphorbiaceae	Euphorbiaceae	13.37	iiP
		Schinus	Anacardiaceae	9.79	iiP
		Miconia	Melastomataceae	7.60	iiP
		Mimosa pudica	Fabaceae	3.52	iiP
		Schinus sp2	Anacardiaceae	2.95	ioP
		Ficus	Moraceae	2.95	ioP
		Asteraceae sp2	Asteraceae	2.26	ioP
		Cecropia	Urticaceae	2.20	ioP
		Elephantopus	Asteraceae	2.07	ioP
		Citrus	Rutaceae	1.95	ioP
		Venonanthura sp2	Asteraceae	1.69	ioP
		Syagrus	Arecaceae	1.63	ioP
		Commelinaceae	Commelinaceae	1.57	ioP
		Poaceae	Poaceae	1.57	ioP
		Croton	Euphorbiaceae	1.32	ioP
		Mimosa sp1	Fabaceae	1.13	ioP
		Asteraceae sp3	Asteraceae	0.82	ioP
		Protium	Burseraceae	0.69	ioP
		Acacia	Fabaceae	0.63	ioP
		Borreria	Rubiaceae	0.56	ioP
		Machaerium	Fabaceae	0.50	ioP
		Inga	Fabaceae	0.44	ioP
		Psychotria	Rubiaceae	0.44	ioP
		Sapium	Euphorbiaceae	0.31	ioP
		Asteraceae sp1	Asteraceae	0.19	ioP
		Mimosa sp2	Fabaceae	0.13	ioP
		Venonanthura sp1	Asteraceae	0.06	ioP

*Classification: DP-dominant pollen (>45% of the total grains in the sample); AP-accessory pollen (16 to 45%); iiP-important isolated pollen (3 to 15%); oiP-occasional isolated pollen (<3%).
